# Effects of erythropoietin on bile duct ligation-induced neuro-inflammation in male rats

**DOI:** 10.3934/Neuroscience.2019.2.43

**Published:** 2019-04-29

**Authors:** Moazameh Golshani, Mohsen Basiri, Mohammad Shabani, Iraj Aghaei, Majid Asadi-Shekaari

**Affiliations:** 1 Department of Anatomical Sciences, Afzalipour School of Medicine, Kerman University of Medical Sciences, Kerman, Iran; 2 Neuroscience Research Center, Neuropharmacology Institute, Kerman University of Medical Sciences, Kerman, Iran; 3 Neuroscience Research Center, Poursina Hospital, Guilan University of Medical Sciences, Rasht, Iran

**Keywords:** bile duct ligation, hepatic encephalopathy, erythropoietin, astrocytes, microglia

## Abstract

Hepatic encephalopathy (HE) is a brain disorder as a result of liver failure. Previous studies have indicated that erythropoietin (EPO) has neuroprotective effects in different neurological diseases. This study addressed the therapeutic effect of a four-week treatment with EPO on neuronal damages in bile duct-ligated rats. Forty male Wistar rats (250–280 g) were used in the present study. The animals were randomly divided into four groups consisting of 10 animals each, including sham, sham + EPO, bile duct ligation (BDL), and BDL + EPO. EPO was intraperitoneally administered every other day (5,000 U/Kg) in the last four weeks after BDL. Biochemical and histological studies were performed to evaluate neurodegeneration. The results revealed that BDL increases the level of hepatic enzymes and total bilirubin. Furthermore, neurodegeneration was significantly increased in the BDL group compared to sham groups. EPO preserved hepatic enzymes and total bilirubin in the treated group. In addition, EPO significantly decreased the neurodegeneration in BDL + EPO compared to the BDL group. Results of this study showed that EPO has neuroprotective effects in the rat model of HE, possibly due to its anti-inflammatory and anti-oxidant properties. Complementary studies are required to clarify the exact mechanisms.

## 1. Introduction

The liver plays an important role in the regulation of glycogen storage, synthesis of protein, and detoxification of various metabolites. Liver failure affects brain function and can lead to neurological and psychological changes, referred to as hepatic encephalopathy (HE). Malfunction of the liver can lead to accumulation of toxic substances such as bilirubin, urea, and ammonium in the blood [1],[2]. Liver failure, hepatitis, cirrhosis, or bile duct obstruction are major factors in HE [3].

The clinical symptoms of HE depend on the rate at which liver dysfunction occurs and also the degree of metabolic disorders [4]. As the disease progresses, motor function and mental abilities become impaired. Patients show a reduced ability in terms of attention, learning, memory, and cognition, and may also suffer from motion and visual perception impairment [5],[6]. These changes affect the quality of life in patients with HE and can lead to impairment in performing daily activities [7].

Hyperammonemia is a major factor in the pathogenesis of HE [8]. Toxins such as ammonia can enter the nervous system. The only way to detoxify ammonia is its conversion to glutamine, and the enzymes of this process can be found only in astrocytes. Product glutamine is transferred to neurons [9]. Increase in glutamate in the brain is characteristic of HE [10],[11].

Furthermore, blocking the flow of bile from the liver to the intestine is associated with profound metabolic changes, including changes in the mitochondrial functions, reduction in fatty acid oxidation, and increases in the concentration of hydrophobic bile acids in the liver and plasma. These factors eventually cause an inflammatory response associated with the death of hepatocytes or cirrhosis [12],[13]. Thus, bile flow obstruction can cause liver failure and, subsequently, HE [3]. By changing the metabolism of neurotransmitters and induction of neurotoxicity, ammonia plays a major role in the development of HE [14].

Previous scientific reports have shown that hyper-ammonia with bile duct ligation (BDL) can lead to intense activity of glial cells in the cerebellum and some areas of the hippocampus [15]. Activation of microglia is associated with the release of inflammatory factors and an increase in the concentrations of pro-inflammatory cytokines in the brain, followed by the severe swelling of astrocytes and brain edema which refers to HE [16],[17]. Different agents such as EPO [18] and pioglitazone [19] are effective against neuronal impairments observed in BDL rats. Interestingly, macrophages expressing EPO receptors and EPO have been reported to suppress inflammatory macrophage activation [20]. EPO is a neuroprotective agent acting by the inhibition of neuronal apoptosis and reduction of neuroinflammation [21].

In our previous studies, we found that BDL rats manifest motor and spatial learning and memory impairments, and chronic treatment with EPO alleviates these impairments [18]. In the present study, the effect of EPO on neuronal degeneration and astrocyte and microglia activity was evaluated in the CA1 subfield of the hippocampus and cerebellum of male rats following common BDL.

## 2. Materials and methods

In the present study, male Wistar rats (n = 40, 2 months old, 250–280 g) were used. The handling and care of the animals were conducted according to the National Guidelines on Animal Care and was approved by the Ethics Committee of the Kerman University of Medical Sciences (Ethics Code: IR.KMU.REC.1396.1140.). Animals were kept under standard conditions; room temperature was controlled (20 ± 2 °C) and a 12h on-off light-dark cycle was maintained with free access to water and food. The rats were randomly divided into four groups consisting of 10 animals in each group (sham surgery, sham surgery + EPO, BDL surgery, and BDL surgery + EPO). EPO was prepared from Pooyesh Darou Product Company and intraperitoneally administered every other day (5,000 U/Kg) [i.p.] in the final four weeks after BDL [18].

Sham and BDL surgery groups received saline intraperitoneally in the same volume and based on the time schedule considered for EPO groups. All experimental studies were performed in a blind manner.

### 2.1. BDL procedure

Rats were anaesthetized (ketamine 90 mg/kg, xylazine 12 mg/kg, i.p.). A middle abdominal incision was performed. Then, after cutting the fascia and muscles, the common bile duct was ligated with a 4–0 silk suture at two points posterior to the hilum of the liver and anterior to the pancreas. The abdominal incision was closed in two layers. In sham animals, the common bile duct was manipulated but not ligated. All animals were maintained for six weeks following the surgery [4],[22]. The mortality rate was 20% in the BDL group and 5% in the BDL surgery + EPO, and new animals were replaced.

### 2.2. Biochemical and cytological parameters

Animals were sacrificed under deep anesthesia at the end of the sixth week of surgery and blood samples were collected by carotid bleeding. The following parameters were biochemically assayed in the plasma separated from blood samples: total bilirubin (Sigma, MAK126), alkaline phosphatase (ALP) (Sigma, APF), Aspartate transaminase (AST) (Sigma, MAK055) and hepatic albumin (Sigma, MAK124) using a commercially available kit. In addition, cytological parameters, consisting of red blood cells (RBCs) and hemoglobin were evaluated [23],[24].

### 2.3. Tissue preparation

Animals were sacrificed under deep anesthesia six weeks after BDL and their brain was removed and dissected into hippocampal and cerebellum blocks. The brains were fixed in a 10% formalin solution for 48 h and processed for histological and immunohistochemistry (IHC) analysis.

### 2.4. Nissl staining

To assess the morphology of hippocampus and cerebellum neurons, the Nissl staining method was employed. Coronal sections (8 µm) were cut from the hippocampus and cerebellum using a rotary microtome. Briefly, sections were deparaffinized through xylene and alcohols into tap water, stained in 0.1% cresyl violet solution for 5 min, dehydrated in 100% alcohol, and then cleared in xylene. Pyramidal neurons of the CA1 sector of the hippocampus and Purkinje cells of the cerebellum were manually counted in three microscopic fields (0.107 mm^2^; 89.82 × 120.70 µm) of the Nissl stained sections from the hippocampus and cerebellum. Results are expressed as the average number of cells/0.1 mm^2^.

### 2.5. Immunohistochemistry

Astrocyte glial fibrillary acidic protein (GFAP) and microglia (CD11) were counted in order to evaluate astrocyte and microglial activity. The sections were immunostained using the polyclonal primary antibody for GFAP (1:400) (PA1239) or CD11 (1:500) (DB Biotech).

The sections were deparaffinized in xylene, hydrated through a graded ethanol series, and washed in running water. After antigen retrieval, the sections were incubated with rabbit primary antibody GFAP [25] and mouse primary antibody CD11 [26] overnight at 4 °C. Afterwards, the sections were rinsed for 10 min with phosphate buffer solution (PBS) and incubated with horseradish peroxidase (HRP)-conjugated anti-rabbit and anti-mouse antibodies (Santa Cruz Biotechnology, Inc., USA) at the dilution of 1:200 for 1 h at room temperature. After washing in PBS, peroxidase activity was detected with 3, 3-diaminobenzidine (DAB, Abcam, UK) as the chromogenic substrate. The sections were counterstained with he-matoxylin (Sigma-Aldrich, USA), dehydrated in an increasing alcohol series, cleared in xylene, and finally mounted on Entellan® (Merck, Germany). Astrocytes showing GFAP and CD11^+^ cells were manually counted in three microscopic fields (0.107 mm^2^; 89.82 × 120.70 µm) of the immunohistochemically stained sections from the cerebellum and hippocampus. Results are expressed as the average number of cells/0.1 mm^2^.

### 2.6. Statistical analyses

All statistical analyses were performed using SPSS (v.22). All data were calculated for normality using the Kolmogorov-Smirnov test. Results were normally distributed (*p* < 0.05 in K-S test), and therefore expressed as the mean ± SEM and analyzed using one-way ANOVA. Tukey's post-hoc analysis was used for the analysis of data, and *p* < 0.05 was considered as the significance level.

## 3. Results

### 3.1. Effects of BDL and EPO on biochemical and cytological parameters

The levels of biochemical and cytological parameters were elevated as a result of BDL surgery after six weeks. Based on our data, BDL induction resulted in a significant increase (*p* < 0.05) in ALP and ALT levels and the administration of EPO counteracted these effects. Total bilirubin levels were elevated in BDL animals and were not affected by EPO administration, either in sham or in BDL animals. EPO administration increased the number of RBCs in sham and BDL groups. There was no significant change in the level of hemoglobin across groups. The level of albumin markedly increased in the BDL rats with the EPO treatment compared to BDL (Table 1).

### 3.2. Effect of BDL and EPO on neuronal degeneration

**Figure 1.**
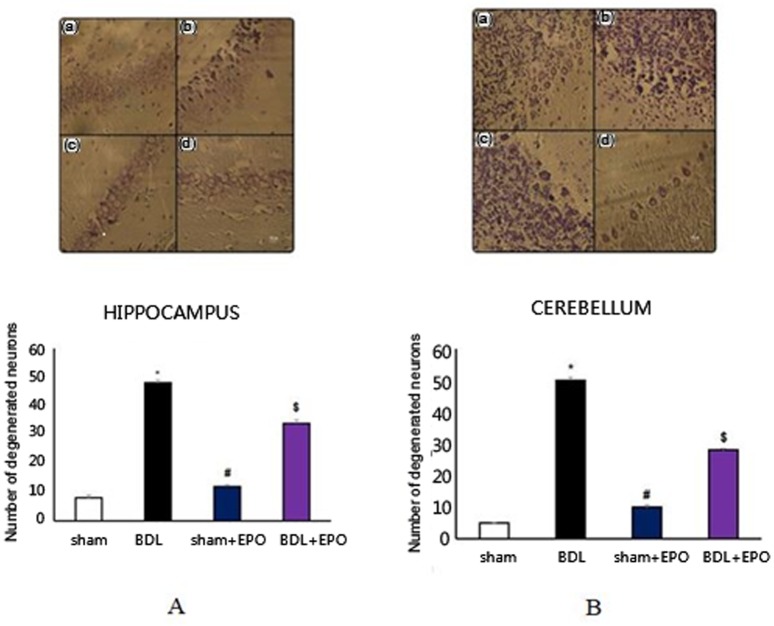
The effects of BDL and EPO treatment on neuronal injury induced by BDL in male rats. Representative photomicrographs showing cerebellar architecture (A) and hippocampus (B) in male rats in different groups: **(a)** sham, **(b)** BDL, **(c)** sham + EPO, **(d)** BDL + EPO. BDL induced insults to cerebellar Purkinje and hippocampal pyramidal neurons and the administration of EPO ameliorated the detrimental effects in treated animals. The figure shows the quantitative analysis of and hippocampal pyramidal neurons cerebellar Purkinje in different groups. Data are the mean ± SEM. *p < 0.001 compared with the sham group; # and $ *p* < 0.001 compared with BDL (one-way ANOVA with Tukey's post-hoc test for all comparisons).

Nissl staining was performed to determine neuronal degeneration. Results showed that BDL significantly increased the number of degenerated neurons in the hippocampus (Figure 1A) and cerebellum (Figure 1B) compared to the shams group. Treatment with EPO significantly decreased BDL-induced neurodegeneration.

**Table 1.** The effects of BDL and EPO treatment on hepatic enzymes, albumin, bilirubin total, hemoglobin, and RBCs.

Groups	AST (U/l)	ALP (U/l)	Albumin (g/dl)	Bilirubin total (mg/dl)	RBC (number × 106 ul)	Hb (g/dl)
Sham	598 ± 88.7	416 ± 7.23	3.40 ± 0.3	0.4 ± 0.1	5.53 ± 0.07	11.5 ± 0.3
BDL	1138 ± 24.99*	613 ± 5.69*	1.90 ± 0.06*	8.5 ± 0.26*	4.5 ± 0.21	10.6 ± 0.33
Sham + EPO	481 ± 5.11^$^	329 ± 3.79^$^	3.97 ± 0.08^$^	0.76 ± 0.02^$^	9.66 ± 0.27^$^	13.7 ± 0.31
BDL + EPO	845 ± 8.41^#^	531 ± 4.77^#^	3.06 ± 0.04^#^	9.4 ± 0.29	6.5 ± 0.16	11.8 ± 0.07

Note: BDL significantly increased the level of AST and ALP. The level of total bilirubin was significantly increased in BDL rats. Albumin level was decreased in BDL and BDL+EPO rats. Moreover, EPO treatment reduced AST and ALP levels. BDL did not significantly change the RBC parameters, although EPO increased these parameters. *p < 0.05, compared with the sham; $ and # *p* < 0.05, compared with the BDL.

### 3.3. Effect of BDL and EPO on glial cells

**Figure 2.**
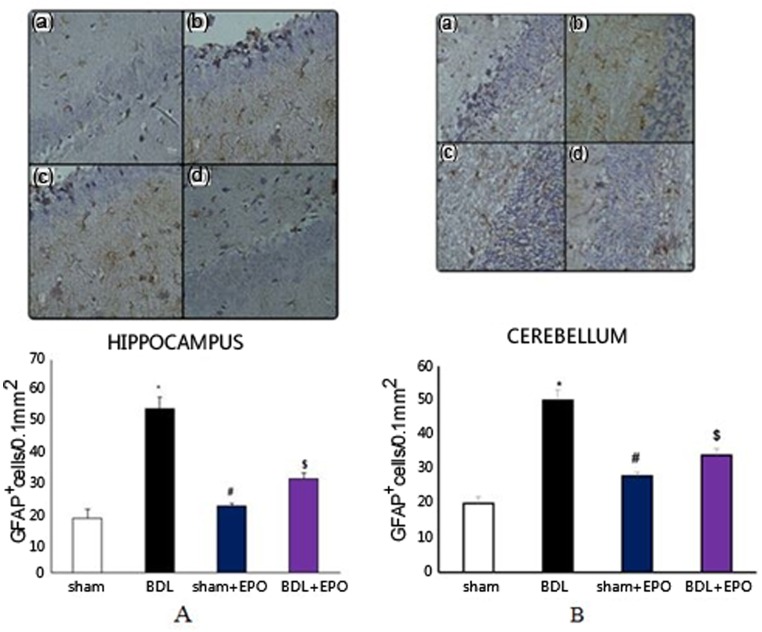
The effect of BDL and EPO treatment on the number of GFAP^+^ cells in the hippocampus (A) and cerebellum (B) of rats. Representative photomicrographs showing GFAP^+^ cell morphology after staining using antibodies against GFAP in different groups of rats: **(a)** sham, **(b)** BDL, **(c)** sham + EPO, **(d)** BDL + EPO. Nuclei were stained with hematoxylin (blue). Magnification 400×. The graph shows the quantitative analysis of GFAP^+^ cells in the hippocampus and cerebellum of rats in different groups. Data are the mean ± SEM. *p < 0.001 compared with the sham group; # and $ (*p* < 0.001 and 0.1, respectively) compared with BDL (one-way ANOVA with Tukey's post-hoc test for all comparisons).

Differences in the number of GFAP^+^ and CD11^+^ cells between the different groups in the hippocampus and cerebellum were analyzed using one-way ANOVA. Results demonstrated that the number of GFAP^+^ cells in the BDL group was significantly higher than the sham and sham + EPO groups (*p* < 0.001) in the hippocampus (Figure 2A) and cerebellum (Figure 2B), whereas EPO treatment significantly decreased cell number in the treated group (*p* < 0.01).

**Figure 3.**
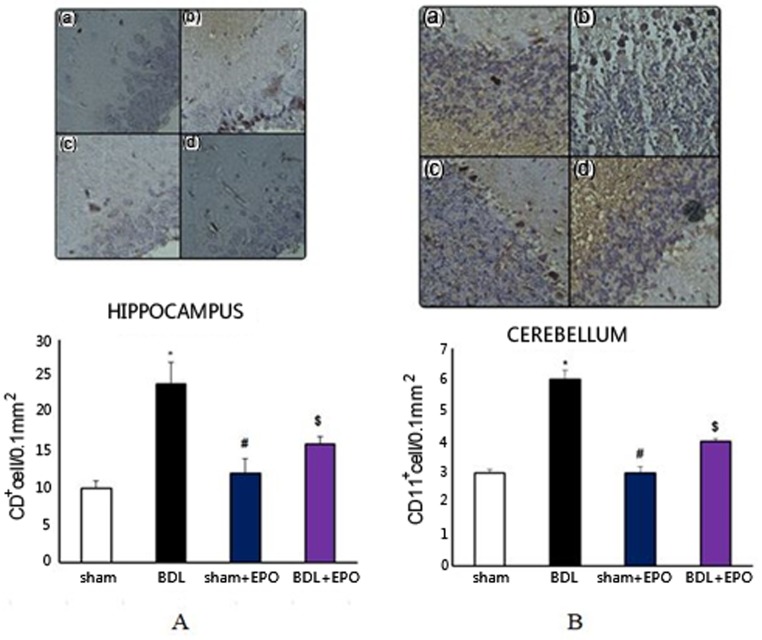
The effect of BDL and EPO treatment on the densitometry of CD11^+^ cells in the (A) hippocampus and cerebellum (B) of rats. Representative photomicrographs showing CD11^+^ cell morphology after staining using antibodies against CD11 in different groups of rats: **(a)** sham, **(b)** BDL, **(c)** sham + EPO, **(d)** BDL + EPO. Nuclei were stained with hematoxylin (blue). Magnification 400×. The graph shows the quantitative analysis of CD11^+^ cells of hippocampus and cerebellum in different groups. Data are the mean ± SEM. *p < 0.01 compared with the sham group; # and $ *p* < 0.01 compared with BDL (one-way ANOVA with Tukey's post-hoc test for all comparisons).

In addition, the data demonstrated that the number of CD11^+^ cells was significantly higher in the BDL than sham and sham + EPO (*p* < 0.001) groups, whereas cell number was significantly decreased in the EPO treated group (*p* < 0.01, Figure 3A and 3B).

## 4. Discussion

Previous studies have demonstrated deficits in motor and cognitive in both clinical and animal experiments of HE following BDL [18],[27],[28]. Previously, the possible effect of EPO as a potent neuroprotective agent on motor and cognitive impairments induced by HE were also identified. In the present study, the effects of EPO on the neuronal degeneration and astrocyte and microglia activity of male rats following BDL were investigated. Deficits in liver function and neuronal degeneration were observed in rats following BDL as an animal model of HE. EPO had a neuroprotective function against hepatic enzyme impairments and neurodegeneration induced by BDL.

Although the exact mechanisms of impairments are not yet clear, neural apoptosis, oxidative stress, and excitotoxicity have been proposed to underlie these impairments [1]. Increased levels of free radicals have been observed in the brain of BDL rats [29]. Therefore, finding strategies to decrease these oxidative changes may help reduce the impairments observed in HE.

EPO is responsible for RBC production stimulation in the bone marrow. It has been demonstrated that EPO can be a potent neuroprotective agent [1]. BDL is known to induce neural damage through oxidative stress and metabolic imbalance [29]. Therefore, it appears that EPO may exert its effect on BDL-induced impairments through these pathways.

In agreement with a previous study, BDL induced a significant increase in the bilirubin level of BDL rats [30], and EPO did not alter this biochemical change in the male rats. This may imply that EPO protects neurons from other pathways [31]. Additionally, as a result of the present study, BDL-induction resulted in a decrease in albumin, and EPO administration reversed the effect of BDL. Thus, EPO may protect neurons through its albumin increasing activity.

Albumin provides more than 50% of the total antioxidant of the plasma. The activity is attributed to the abundant reduced sulfhydryl groups of albumin that can scavenge a variety of free radicals such as nitric oxide and hypochlorous acid. On the other hand, albumin can bind to unconjugated bilirubin, a potent antioxidant. It is believed that this antioxidant activity is usually assumed to be the mechanism responsible for the potent inverse correlation between plasma unconjugated bilirubin concentration and mortality of many diseases [32].

In the present study, the data demonstrated that EPO ameliorates biochemical impairments induced by BDL. Caillaud et al. showed that EPO reduces the systematic oxidative stress in the treated rats [33]. Consequently, the possible mechanism of action for EPO may be through its antioxidant properties.

Previous studies have shown that BDL can lead to neuronal degeneration and activation of astrocytes and microglia in the hippocampus and cerebellum [21],[34]–[36]. Several studies have suggested that EPO, as an internal mediator, has significant neuroprotective effects in various disorders of the central nervous system (CNS) [37]. It possibly acts as a neuroprotective agent in the treatment of neurodegenerative diseases by modulating inflammatory factors [38]–[40]. Bond et al. reported that, through the reduction of reactive oxygen species (ROS) levels, EPO inhibits neuroinflammation and neuronal death [21].

In this study, the results revealed an increase in GFAP^+^ and CD11^+^ cells in the cerebellum and hippocampus following BDL, and the number of these cells showed more astrocytes compared to microglia cells in these areas. Microglia cells are known as the first line of defense against CNS disorders and injuries, including stroke, brain injury, and spinal cord injury [41]. There is extensive evidence that microglia cells have an important role in CNS homeostasis and its related damage. Following damage, microglia cells quickly migrate to the lesion site and prevent long-term lesion progress [41]. Activation of microglial cells is an important part of neuroinflammation, widely discussed in HE. Astrocytes are known as key cells in brain damage due to acute liver disease, but there is evidence that microglia cells are equally involved in neuroinflammation as a result of acute liver disease [42].

Jayakumar et al. showed that hyperammonemia following HE leads to microglia (mainly in the cerebellum) and brain endothelial cell activation that is associated with the production and release of inflammatory factors as a result of which astrocytic swelling and cerebral edema occurs [16].

Bond et al. showed that, through decreasing the level of ROS and reactive nitrogen species, EPO limits the microglia and astrocytes infiltration. On the other hand, EPO can maintain the health of endothelial cells of the blood-brain barrier [21].

## 5. Conclusion

In conclusion, our results demonstrated that EPO administration can reduce cholestasis-induced liver dysfunction. It also reduces neurodegeneration in the hippocampus and cerebellum following BDL. Additionally, EPO modulates the number of GFAP^+^ and CD11^+^ cells in BDL groups. These effects are probably related to the potential anti-oxidant and anti-inflammatory effect of EPO. However, further studies are needed to prove this hypothesis.

## References

[1] Felipo V (2013). Hepatic encephalopathy: effects of liver failure on brain function. Nat Rev Neurosci.

[2] Olde Damink SW, Jalan R, Dejong CH (2009). Interorgan ammonia trafficking in liver disease. Metab Brain Dis.

[3] Butterworth RF, Norenberg MD, Felipo V (2009). Experimental models of hepatic encephalopathy: ISHEN guidelines. Liver Int.

[4] Leke R, Oliveira DL, Forgiarini LF (2013). Impairment of short term memory in rats with hepatic encephalopathy due to bile duct ligation. Metab Brain Dis.

[5] Weissenborn K, Giewekemeyer K, Heidenreich S (2005). Attention, memory, and cognitive function in hepatic encephalopathy. Metab Brain Dis.

[6] Ferenci P, Lockwood A, Mullen K (2002). Hepatic encephalopathy--definition, nomenclature, diagnosis, and quantification: final report of the working party at the 11th World Congresses of Gastroenterology, Vienna, 1998. Hepatology.

[7] Patel A, Wade JB, Thacker LR (2014). 991 Brain Reserve Modulates Health-Related Quality of Life in Patients With Cirrhosis Independent of Covert Hepatic Encephalopathy and MELD Score. Gastroenterology.

[8] Rose CF (2012). Ammonia-lowering strategies for the treatment of hepatic encephalopathy. Clin Pharmacol Ther.

[9] Savlan I, Liakina V, Valantinas J (2014). Concise review of current concepts on nomenclature and pathophysiology of hepatic encephalopathy. Medicina (Kaunas).

[10] Rama Rao KV, Jayakumar AR, Norenberg MD (2012). Glutamine in the pathogenesis of acute hepatic encephalopathy. Neurochem Int.

[11] Rothman DL, De Feyter HM, Maciejewski PK (2012). Is there in vivo evidence for amino acid shuttles carrying ammonia from neurons to astrocytes?. Neurochem Res.

[12] Jover R, Rodrigo R, Felipo V (2006). Brain edema and inflammatory activation in bile duct ligated rats with diet-induced hyperammonemia: A model of hepatic encephalopathy in cirrhosis. Hepatology.

[13] Sheen JM, Huang LT, Hsieh CS (2010). Bile duct ligation in developing rats: temporal progression of liver, kidney, and brain damage. J Pediatr Surg.

[14] Aldridge DR, Tranah EJ, Shawcross DL (2015). Pathogenesis of hepatic encephalopathy: role of ammonia and systemic inflammation. J Clin Exp Hepatol.

[15] Rodrigo R, Cauli O, Gomez-Pinedo U (2010). Hyperammonemia induces neuroinflammation that contributes to cognitive impairment in rats with hepatic encephalopathy. Gastroenterology.

[16] Jayakumar AR, Rama Rao KV, Norenberg MD (2015). Neuroinflammation in hepatic encephalopathy: mechanistic aspects. J Clin Exp Hepatol.

[17] Butterworth RF (2012). Reprint of: Neuroinflammation in acute liver failure: Mechanisms and novel therapeutic targets. Neurochem Int.

[18] Aghaei I, Nazeri M, Shabani M (2015). Erythropoietin ameliorates the motor and cognitive function impairments in a rat model of hepatic cirrhosis. Metab Brain Dis.

[19] Aghaei I, Shabani M, Doustar N (2014). Peroxisome proliferator-activated receptor-gamma activation attenuates motor and cognition impairments induced by bile duct ligation in a rat model of hepatic cirrhosis. Pharmacol Biochem Behav.

[20] Nairz M, Schroll A, Moschen AR (2011). Erythropoietin contrastingly affects bacterial infection and experimental colitis by inhibiting nuclear factor-kappaB-inducible immune pathways. Immunity.

[21] Bond WS, Rex TS (2014). Evidence that erythropoietin modulates neuroinflammation through differential action on neurons, astrocytes, and microglia. Front Immunol.

[22] Aghaei I, Hajali V, Dehpour A (2016). Alterations in the intrinsic electrophysiological properties of Purkinje neurons in a rat model of hepatic encephalopathy: Relative preventing effect of PPARgamma agonist. Brain Res Bull.

[23] Shabani M, Ebrahimpoor F, Firouzjaei MA (2019). Modulation of sphingosine-1-phosphate receptor by FTY720 contributes in improvement of hepatic encephalopathy induced by bile duct ligation. Brain Res Bull.

[24] Tahamtan M, Aghaei I, Pooladvand V (2017). Characterization of the CA1 pyramidal neurons in rat model of hepatic cirrhosis: insights into their electrophysiological properties. Metab Brain Dis.

[25] Onoda A, Takeda K, Umezawa M (2017). Dose-dependent induction of astrocyte activation and reactive astrogliosis in mouse brain following maternal exposure to carbon black nanoparticle. Part Fibre Toxicol.

[26] Matsui H, Ohgomori T, Natori T (2013). Keratan sulfate expression in microglia is diminished in the spinal cord in experimental autoimmune neuritis. Cell Death Dis.

[27] Javadi-Paydar M, Ghiassy B, Ebadian S (2013). Nitric oxide mediates the beneficial effect of chronic naltrexone on cholestasis-induced memory impairment in male rats. Behav Pharmacol.

[28] Nasehi M, Piri M, Abbolhasani K (2013). Involvement of opioidergic and nitrergic systems in memory acquisition and exploratory behaviors in cholestatic mice. Behav Pharmacol.

[29] Butterworth RF (2011). Hepatic encephalopathy: a central neuroinflammatory disorder?. Hepatology.

[30] Huang LT, Tiao MM, Tain YL (2009). Melatonin ameliorates bile duct ligation-induced systemic oxidative stress and spatial memory deficits in developing rats. Pediatr Res.

[31] Brines M, Cerami A (2005). Emerging biological roles for erythropoietin in the nervous system. Nat Rev Neurosci.

[32] Levitt DG, Levitt MD (2016). Human serum albumin homeostasis: a new look at the roles of synthesis, catabolism, renal and gastrointestinal excretion, and the clinical value of serum albumin measurements. Int J Gen Med.

[33] Caillaud C, Mechta M, Ainge H (2015). Chronic erythropoietin treatment improves diet-induced glucose intolerance in rats. J Endocrinol.

[34] Dhanda S, Sandhir R (2015). Role of dopaminergic and serotonergic neurotransmitters in behavioral alterations observed in rodent model of hepatic encephalopathy. Behav Brain Res.

[35] Su YY, Yang GF, Lu GM (2015). PET and MR imaging of neuroinflammation in hepatic encephalopathy. Metab Brain Dis.

[36] Chen JR, Wang BN, Tseng GF (2014). Morphological changes of cortical pyramidal neurons in hepatic encephalopathy. BMC Neurosci.

[37] Ponce LL, Navarro JC, Ahmed O (2013). Erythropoietin neuroprotection with traumatic brain injury. Pathophysiology.

[38] Wenker SD, Chamorro ME, Vittori DC (2013). Protective action of erythropoietin on neuronal damage induced by activated microglia. FEBS J.

[39] McPherson RJ, Juul SE (2008). Recent trends in erythropoietin-mediated neuroprotection. Int J Dev Neurosci.

[40] Mofidi A, Bader A, Pavlica S (2011). The use of erythropoietin and its derivatives to treat spinal cord injury. Mini Rev Med Chem.

[41] Hu X, Liou AK, Leak RK (2014). Neurobiology of microglial action in CNS injuries: receptor-mediated signaling mechanisms and functional roles. Prog Neurobiol.

[42] Wright G, Swain M, Annane D (2016). Neuroinflammation in liver disease: sessional talks from ISHEN. Metab Brain Dis.

